# Lived experiences of radiation therapists using health literacy strategies with patients—A qualitative review using interpretative phenomenological analysis

**DOI:** 10.1002/jmrs.574

**Published:** 2022-03-14

**Authors:** Toni Kelly, Jennifer D Thompson, Yolanda Surjan, Marianne Rinks, Helen Warren‐Forward

**Affiliations:** ^1^ School of Health Sciences University of Newcastle Callaghan New South Wales Australia; ^2^ 226154 Illawarra Shoalhaven Local Health District Wollongong New South Wales Australia; ^3^ BreastScreen New South Wales, Hunter New England Newcastle New South Wales Australia

**Keywords:** Cancer < General, Radiation Oncology < Discipline, Research–qualitative < General

## Abstract

**Introduction:**

Adaptive communication is an essential requirement to deliver quality patient‐centred care. Determining patients’ informational needs and acting on the needs are skills radiation therapists (RTs) employ daily with patients. Learning health literacy (HL) strategies to assist with the informational delivery provides RTs with options to improve patients’ understanding of vital radiotherapy treatment information or tasks. This research investigates the lived experiences of RTs from the Illawarra and Shoalhaven Cancer Care Centres in Australia using HL strategies during patient interactions after undertaking HL training workshops.

**Methods:**

An interpretative phenomenological analysis (IPA) approach was used. Audio‐recorded semi‐structured interviews were conducted with six RTs. Two reviewers analysed each interview script separately before discussing and constructing substantive and sub‐themes.

**Results:**

Four substantive themes were constructed: RT personal attitudes and responses to HL, HL strategies used by RTs, patient associated HL needs and barriers when addressing patient HL needs. RTs were either person‐ or process‐focussed during patient interactions. It was identified that information is provided to patients according to how RTs themselves like to learn new information.

**Conclusion:**

This research has allowed an opportunity to inquire into the lived experiences of RTs implementing HL strategies when providing information to patients. While RTs may be person or process‐focussed, the patient’s needs are always prioritised when providing information, which ultimately results in patient understanding and increased engagement.

## Graphical abstract

Determining patients’ informational needs and acting on the needs are skills radiation therapists (RTs) employ daily with patients. Learning health literacy tools to assist with the informational delivery provides RTs with options to improve patients’ understanding of vital radiotherapy treatment information or tasks. While RTs may be person or process‐focused when interacting with patients, the patient’s needs are always prioritised when providing information which ultimately results in patient understanding and increased compliance.

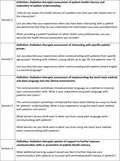

## Introduction

Effective patient–health professional partnerships are derived through a patient‐centred care approach.^1^, ^2^ Highly adaptive communication skills of healthcare providers (HCP) must first determine the patient's informational needs and then manage those needs.^3^ A patient's inability to understand health information can hinge on a range of factors. Anxiety around receiving a life‐threatening diagnosis^4^ such as cancer and physical and emotional problems related to previous trauma^4^, ^5^ are two examples a person may not take on, process or understand new information.

Health literacy (HL) describes how people access, understand, appraise and apply health information in order to make decisions about their health.^3^ In Australia, 60% of people have limited HL^3^ based on the 2006 Australian Bureau of Statistics (ABS).^3^ In 2018, the ABS used results from the Australian Health Survey to classify 44 items into nine domains of a HL assessment.^6^ New domains specific to the vital role of HCPs included feeling understood and supported by HCPs, having sufficient information to manage their health, and actively engaging with HCPs.^6^

The expected patient benefits when radiation therapists (RTs) engage in HL strategies with patients allow the identification of psychosocial issues and, in relation to radiotherapy procedures, an opportunity for patients to seek clarity to treatment and to improve management of treatment side effects at home.^7^ Smith et al.^2^ explored Australian RTs perspectives on supporting people with limited HL undergoing radiotherapy, resulting in guidelines for RTs to use with patients with different levels of HL. Montgomery et al.^7^ provided recommendations for identifying radiotherapy patients at risk of limited HL from a Canadian perspective and developed communication strategies designed for patients with limited HL. Schnitzler et al.^8^ recommend routinely checking for understanding during consultations, as patients may not always understand information provided. Quinn et al.^9^ determined Irish RTs with little HL knowledge, education or training could recognise patients with limited levels of HL. Quinn^9^ identified strategies providing support for patients with limited HL and recommendations including RT staff awareness, the development of patient aids and screening. A gap in the literature exists of experiences of RTs who have undertaken specific communication and HL training and then used these new HL strategies with patients.

This research expands on previous outcomes from communication and HL skills workshops delivered to RTs at the Illawarra and Shoalhaven Cancer Care Centres.^10^, ^11^ Participating RTs completed three surveys at three time points (pre‐, post‐ and three months post‐workshops) and were evaluated both quantitatively and qualitatively. The use of plain language with patients at three months post‐survey was significantly higher than the use of the teach‐back method.^11^ Plain language is language delivered at a 12‐ to 14‐year‐old level.^3^ The teach‐back method asks the patient to repeat back in their own words what they need to know to check for understanding.^3^ Resultant RT themes from the qualitative review were *improved patient understanding, impact on professional credibility*, *practice and timing of using methods*, *appearing to be condescending to educated patients* and *increased patient anxiety*.^10^ Two themes, *impact on professional credibility* and *appearing to be condescending to educated patients,* were unexpected outcomes, and the authors wanted to delve deeper into these phenomena.

As a follow on, this research aimed to explore the lived experiences of RTs using HL strategies learnt in communication and HL skill training workshops with patients during their daily interactions.

## Methodology

### Theoretical approach

This study seeks to understand RT experiences of employing HL strategies with patients using interpretive phenomenological analysis (IPA).^12^ An IPA study interprets how people try to make sense of their experiences.^12^ IPA applies very small, purposive sampling techniques where participants are selected based on similar experiences^12^ and centres on a double hermeneutic (interpretation) approach. The researcher interprets the participant account of the phenomenon, where the participant is trying to interpret their own experiences. A systematic and self‐conscious approach is applied by the researcher using the participant’s own account of their experiences to construct substantive and sub‐themes though transcript analysis.^12^ This qualitative research approach allows the researcher to delve deeper into participants’ experiences than can be retrieved through survey analysis alone.

### Participants and recruitment

Eligibility criteria to participate centred on RTs completing two communication and HL workshops in early 2018. The research was advertised via a group e‐mail containing a participation information sheet and a consent form.

Six participants were interviewed (Table 1) in May 2021, three years after the workshops. This time delay can determine what strategies participants used from the workshops and continue to implement.

**Table 1:** Participant demographics.

Participant	Gender	Age Range (Years)	Professional Experience (Years)	Interview time (Minutes)
1	Male	45–50	13	45.18
2	Female	20–25	4	60.27
3	Male	30–35	5	40.22
4	Female	40–45	18	18.45
5	Female	35–40	17	18.44
6	Female	40–45	21	25.52

### Interview design

A semi‐structured interview technique was employed. Interview questions (Table 2) were developed in response to the outcomes of the previous research and provided to participants four hours before the interview. Fourteen questions were divided into four domains as defined in Table 2.

**Table 2:** Interview questions sample.

Domain 1	**Definition: Radiation therapist assessment of patient Health Literacy and estimation of patient understanding**. How do you assess the health literacy of a patient you have just met, based only on the interaction? Can you describe your experiences when you have been interacting with a patient and determine that they do not understand the information you have provided them. When providing a patient handover to other health care professionals, can you describe the health literacy assessment you provide?
Domain 2	**Definition: Radiation therapist assessment of interacting with specific patient groups** Can you describe your experiences when communicating with patients from specific age groups? Starting with children, young adults up to age 20 and patients over 70. Can you describe your experiences when communicating with patients where English is a second language?
Domain 3	**Definition: Radiation therapist assessment of implementing the teach‐back method and plain language into the clinical environment**. The communication workshops introduced plain language as a method to improve your communication skills. What is your experience using plain language with patients and carers? The communication workshops introduced the teach‐back method as a way to check for patients’ understanding. What is your experience using the teach‐back method with patients and carers? What barriers do you think exist to deter you from using plain language when communicating with patients? What barriers do you think exist to deter you from using the teach‐back method when communicating with patients?
Domain 4	**Definition: Radiation therapist opinion of support to further improve communication skills or promotion of patient Health Literacy**. What additional learning support would you like to further improve your communication with patients or to assist with promoting health literacy in patients?

The interviews were conducted by the primary researcher (TK) either in person or over Skype (Microsoft^©^, 2021). Only the interviewer and the interviewee were present, and the interviews were digitally recorded, allowing the verbal data to be transcribed and non‐verbal behaviour to be observed and recorded. It is acknowledged that non‐verbal cues were limited for the interview conducted via Skype.

### Data analysis

Data were analysed independently by two reviewers using IPA guidelines specified by Smith et al.^12^ One reviewer was the RT who conducted the interviews and has some experience in qualitative analysis, and the second was an experienced qualitative researcher. Descriptive comments were made with a focus on what specifically was said. Linguistic comments were developed by reviewing specific use of language and conceptual comments evolved by interrogating the data with an element of personal reflection.^12^ Notes were made in a margin of the RTs making sense of the processes they use to ensure patients understand the information they provide. After reviewing the notes, themes were recorded in another margin. Robust discussions were undertaken between the two reviewers to ensure consistent intersubjective interpretations in the construction of the final substantive and sub‐themes. While it was the intent to only interview six participants—as consistent with IPA methodology, data saturation was seen to occur as participants five and six added nothing different to the other participants.

#### Ethics

Ethics approval was sought from the Hunter New England Human Research Ethics Committee (2019/ETH08846).

#### Rigour

Credibility^13^ was assessed through member checking of transcripts and dependability^13^ of the research was ensured through using two independent reviewers. Confirmability^13^ was ensured through the primary researcher creating a memo before and during the interview process to bracket out thoughts, opinions and perceptions around previous research outcomes and what outcomes they may wish to arise from this study. An excerpt can be found in Supporting Information.

It should be acknowledged the primary researcher was a Senior in RT in the same department and one of the facilitators of the workshops. As such, there may be issues around authority and power that may have influenced participants' responses.

## Findings and Discussion

Four substantive themes were constructed during the analysis:

- Radiation therapist’s personal attitudes and responses to health literacy
- Health literacy strategies used by radiation therapists
- Patient associated health literacy needs
- Barriers when addressing patient health literacy needs.

### Theme 1: Radiation therapists’ personal attitudes and responses to health literacy

This theme encompassed several sub‐themes, including RTs as learners, experiences and processes, emotions, time and hierarchy of the department (Table 3).

**Table 3:** Radiation therapist personal responses to HL.

3.1 As learners	*‘Yeah, I don't care. How I look isn’t as much of an issue as long as they feel comfortable with me and as long as they don't lose faith in me. As long as they're confident I'm treating them correctly because they can't they can't feel anything. They can't see anything, they have blind faith in us. As long as I keep the faith in us, as long as it doesn't break that then I'm fine with looking however I look, you know’* (P2)
3.2 Experience/Process	*‘I'll approach it with a structure just to break down into different sections so that I can remember what we're talking about. So, for instance, pre‐treatment, going through their expectations of what they're here for? Bringing printed materials are pretty handy. So just discussing this is what you know’* (P3) *‘I don't really consciously think about it that I would do it. I kind of have you know, you have in your head your speech that you're going to say and now as I'm speaking to them and yet assessing if they are understanding or where they're at it will either change my language* (P4)
3.3 Emotions (self and patient)	*‘Depends on the state, the physical and mental state of mind of the person. Yeah, the age, and a lot of other factors*’. (P1) *‘I do get emotional talking to younger patients. I don't know if it's just like the mother in me, I don't step away from talking to the younger ones, but I definitely find it easier and more joyful talking to the older patient’*. (P6)
3.4 Time	*‘I think with PEARL (*personalised education demonstration software*) coming in will be good because I will have so much more time, because if it’s in place, it'll basically replace the day one chat so you can sit there for an hour with them and their family and just going through every possible question they have and really highlighting the importance of bladder prep and the importance of whatever they're doing. They can really take as long as they want. So they're not holding up a process*’ (P2)
3.5 Team player	*‘In CTSIM, we will go to the machine staff and say ‘we've just done this patient, and they've had this issue with us. We anticipate you may have problems with them in the future’. So that's why they're all aware what's going on. So it's easier for them to identify on day one as well that there will be issues so they can they can sort of work around it’* (P5)

The way RTs learn new information themselves impacted the way they educated their patients ‘*I would use visual aids. I like to get something that I can show to explain it visually… I am a very visual person, especially when I'm learning something new. I appreciate that visual clue’* (P6).

The RTs were either more person‐focused (intuitive and addressed learning needs) or more process‐focussed (acted in patient interest, providing minimal information to continue with the task). The exception was P2 who demonstrated both traits. Those who were more person‐focused appeared adaptable to the immediate learning needs of the patient

they want to feel like they're still in control of their own life, …that they are actually resolving the situation, resolving problems, dealing with issues of day to day and dealing with issues of making decisions. And so, it's important to kind of like address them.

Those who appeared more process‐focussed ensured the patient received the information *‘I'll try and break it up into sections that I think makes sense. So, we might go into talking about what we're doing today, then go into, say, the side effects, then talk about treatment rather than jumping around’* (P4).

All RTs prioritised the importance of building rapport with patients in the early stages of their interactions.

And we just kind of like found that little common ground. That was my way of a kind of a built‐up trust and rapport … and be able to move on to the next step. (P1)

Participants revealed that their patient interactions sparked a range of emotions, from joy to empathy and sadness. Participants could determine why patients may not understand information, including a cancer diagnosis, a life‐changing experience, emotional reactions to the situation they find themselves in, their age and grief or trauma. As a result, all participants demonstrated varying levels of emotional maturity and emotional intelligence and were able to adapt to the changing situation of the patient.

The concept of time was strongly noted, with all participants willing to invest time to provide a quality experience to the patient. Quality time invested with the patient at the beginning of technical processes, such as CT scanning day or Day 1 of treatment was thought to have positive consequences for engagement and achieving required outcomes.

Participant responses were strongly indicative of a patient‐centred care approach to providing and assessing patient understanding. The process‐focused staff may have acted in the patient's interests regarding the patient's current emotional or mental state, took the most appropriate action for the patient, provided the minimum information and continued with the set task. These findings align with Merchant et al.’s^14^ impact of time, space and technology within Australian radiotherapy departments. Merchant^14^ confirms the impact of limited time and the need to interact with patients, in a process driven environment prior to treatment. While Merchant’s study involved patient interviews to confirm the radiotherapy patient’s experience, such that it was ‘anxiety provoking’ and at times, ‘cold and bleak’, RTs in this study were cognisant of these sentiments and attempted to build rapport and connection towards the psychosocial management of the patient.

### Theme 2: Health literacy strategies used by radiation therapists

Sub‐themes identified include imparting information, information handling, language/communication methods and using the teach‐back method (Table 4).

**Table 4:** HL strategies used by radiation therapists

4.1 Imparting information	*I'll keep the eye contact, because sometimes they don't listen, or can't hear, but they can read your lips. And so I think it's important to do that. And sometimes you gotta use gestures as well to kind of demonstrate what you're describing globally’*. (P1) *‘Don't assume they have health literacy. So I'll use very broad terms, very basic terms to make sure that they can understand processes I'll be going through. So, for instance, if a patient comes in for treatment, I won't go into the exact detail of the machine and how it works. I'm more of giving him an introduction of this is what it appears like’* (P3)
4.2 Information handling	*‘Again, it's more that plain English as well, like I think about my kids and how would they actually understand what we're going on about. So kind of pretend that I'm talking to my kids because that level of intellect, I guess. And then as I said before, if the patient starts talking, if they are more technical than I'll increase my technical, but then get them to make sure they understand as well’*. (P6)
4.3 Language/communication methods	*‘Particularly, if you see that they're glazing over and switching off, I might want to stop at that moment. Rather than charging on and finishing the bit that I had decided I was going to tell them, I'll stop there and you know they are understanding what we're talking about*’. (P4)
4.4 Teach back as a tool	‘*‘They may not have any more space in their memory to memorise what the key points are, you know, so it's very important to use the teach back method, like to make sure that they understood what you said’ (P1)*

Participants revealed that they all use a combination of active learning, active listening, observation, analogies (e.g. comparing the CT scan as sliced bread), gestures (e.g. pointing in the direction of head or feet when requiring the patient to move), visual aids and paper resources when providing new information to patients.

Overall, all participants provide age‐appropriate methods to deliver information as simply as possible, ranging from using a juvenile voice (P3) and plain, age‐appropriate language to children.

it would be a lot more basic and simple terminology. I would pick up on what the parents have been telling the kids. (P5)

For patients over 70 years, participants acknowledged *‘you need to increase the tone of your voice, so I speak a little bit louder. Sometimes you've got to slow, phrase by phrase, and I'll give a pause in between to give time for them to process’ (*P1).

All participants recognise a lot of information is required as patients traverse the radiotherapy process and gauge the level of understanding (at some point) for each patient interaction. Half of the participants actively employed a funnelling technique; with the beginning very broad *‘do you know why you are here?’ (*P3*)* and then becoming more specific as the interaction allowed. Most participants were advocates of splitting information *‘I'll break the information up into small portions’* (P4) then check for the minimum level of understanding before moving on.

If the patient had perceived limited HL, all participants would pass this on both verbally and record this in the patient information system accessed by all HCPs interacting with the patient. Conversely, if the patient was noted to have high HL, all participants would verbally pass this onto their immediate team but were less likely to record this.

Language use for all participants was based on using plain language to simplify technical and medical jargon consistent with Smith et al.^2^ recommendations. The teach‐back method was employed to check for patient understanding and led to more open communication for all participants. Four participants routinely use teach‐back when discussing bladder and bowel preparation. RTs appear to use teach‐back more readily than previously reported^10^ which now more closely aligns to the Canadian study,^15^ where 75% of respondents assessed patient understanding using the teach‐back method.

### Theme 3: Patient associated health literacy needs

This theme encompasses sub‐themes including patient considerations, limited HL influences, time and the impact of patient carers during patient interactions (Table 5). Participants were able to identify themselves as a patient, provide patient autonomy through their interactions, understand that radiation oncology departments are not typical environments and attempt to normalise patients’ thoughts and feelings as they traverse this life‐changing event.

**Table 5:** Patient associated health literacy needs.

5.1 Patient	*‘Just to recognise your own thoughts and feelings and reactions to situations. I know it's quite easy to get caught up in another person's emotions, but that's their journey. Yeah. We're certainly here to support them. We are not here to take over*’ (P3)
5.2 Health Literacy	*‘Some people will be anxious of course. It might not be their health literacy, that's the problem. It's just that they're overwhelmed’*. (P4)
5.3 Time	*‘It's more just that the patient is happy and that they are they're understanding. And I can reiterate and supplement information as treatment goes along. They don't have to be perfect on day one or day two with their side effects. But as long as you know they get it by around two weeks in’*. (P2) *‘I just find engaging with the person directly is probably the best way to find out what they understand. Um, obviously, if there's a bit if there's clear physical barrier, or some sort of mental delay or anything like that. I'll take extra time to discuss that’* (P3)
5.4 Patient Carer	*‘I went to through bowel and bladder prep, did the teach back method with them. He understood everything was fine…, came back the next day and had not followed any of the instruction. So what I did then was bring in the partner, the wife, into the conversation again and did teach back with her. It started to sink in and the patient kept doing the right things from then on, but it did take that extra person to be involved with the conversation’* (P6)

Half of the participants interviewed (P2, P3 and P4) discussed their awareness of the impact anxiety has on patients’ ability to understand new information rather than having perceived limited HL. In contrast, P3 detailed anxious patients only require critical points of information to undertake the procedure. Two participants (P4, P6) discussed the balance between acknowledging the patients emotional state and providing information to meet patients' needs.

All participants determined that to be completely patient‐focussed takes time. Time to provide clear explanations, time to build trust and rapport and time for checking for understanding.

so I've found that especially those patients, just taking that time to let things sink in and then getting them to reiterate what they understand has been helpful. (P3)

While additional patient questions were determined to be time intensive (P6), it was essential to slow down (P1) and provide time for patients with mental and physical barriers (P3).

A range of participant experiences supported the sub‐theme: patient carer. These included the carer providing an interpretation of the information (in language meaningful to the patient) to assist with patient understanding (P2 and P6), valuing the second set of ears (P1), observing them as a patient support person (P2) and an extra person within the interaction that may present with very different HL levels to that of the patient (P4). Acknowledgement of carers’ roles including emotional support, transport for appointments, domestic cleaning duties and food shopping make it necessary for HCPs to continue to consider and manage patient carers needs during radiotherapy interactions.^16^

The interviews raised an important point regarding the realisation of when patients exhibit high levels of anxiety, as patients determined to be in distress are less inclined to absorb important information relating to their treatment.^17^ This is supported by ^18^report emotional elements such as fear, shock and anxiety may prevent patients from engaging with new information.^17^ This was also confirmed by ^19^, where the suggestion is made to engage the patient in their own learning or provide the patient with some control over the situation.^18^ Simply by asking the patient how much information they would prefer to know on the day allows the tailoring of information to the level of detail to match the patient’s expectations.

### Theme 4: Barriers when addressing patient health literacy needs

Barriers encountered were divided into patient‐related and RT‐related barriers. An example of a patient‐related barrier is.

when they've got someone with them, like a family member, and they're of quite different education levels. You might have the carer asking you really complicated questions and wanting complicated, complex answers, but the patient does not quite understand it. So that can be tricky, trying to find that balance of respecting the person asking you questions and giving them an answer in language that they want, but then also saying in a way that the patient understands. (P4)

While this is a challenging situation not reported within the literature, many HCPs encounter it regularly. Osborne^19^ suggests solution for these and other patient‐related barriers. Patient‐related barriers such as high anxiety leading to shutting down (P5), fixation on single aspects of treatment or not understanding their diagnosis align with Lambert et al.^20^ findings. Table 6 shows the patient‐related barriers from this research, and solutions suggested using the literature.^19^, ^21^, ^22^ These are important learnings that can be used to address situational issues with patients to determine a mutually beneficial outcome for the patient and RTs delivering patient‐centred care.

**Table 6:** Tips for addressing patient‐related barriers when using HL strategies with patients.

PATIENT‐RELATED BARRIERS	
Barrier	Option for a solution
Patient and carer (patient and adult child) with different level of HL	Ensure the patient’s informational needs are met first and then carefully adapt language and terminology to the carer without losing or offending the patient.^18^
Patients who have high levels of anxiety may shutdown	Identifying the issue through using emotional cues and be ready to be adaptable.^20^ Ask patients how much they want to know, how much detail, some want a lot, some do not want any. Asking them directly will allow that endpoint to be reached much sooner during conversations. Encouraging a carer or family member to act as an advocate to be a part of the session.^18^
Effect of past trauma for patients undertaking treatment (especially in a hospital setting)	Identifying the issue using emotional cues and be ready to be adaptable.^20^ Use active listening to determine where to pitch procedural information. Ask patients how much they want to know, how much detail, some want a lot, some do not want any. Asking them directly will allow a successful procedure to conclude much sooner during conversations. Do not ask about the past unless the patient raises it. Gauge the use of teach‐back, it may upset/antagonise them.
Patient fixated on one aspect of their treatment	Think about information sources the patient is fixed on and how it may relate to some of their questions. Questions allow people to learn new content and determine their level of understanding.^18^
Patient decision makes RT support more difficult	Respecting the patient’s decision is important. Sometimes we do not always have the full picture to understand patients’ motivations. If it helps, identify the issue to help the patient work through it. Keep attempting to use universal precautions approach to providing information.^21^
Patient perception of plain language—the patient did not understand they had a cancer diagnosis	Beginning with ‘why are you here today?’ may provide a quick pathway to determine where to pitch your information. Use clear, plain language and teach‐back to ensure comprehension.^18^

RT‐related barriers raised included finding the right words to translate technical or medical jargon and managing the informational needs of medical doctors as patients who may have an element of assumed knowledge. Two examples of RT‐related barriers:

Using year eight level of language can be tricky sometimes trying to find the right words to describe things that are quite complicated. I find analogies work quite well’ (P4) and ‘There have been occasions when people, like a doctor, you feel you need to elevate this language that you are using because they tend to feel like you're dumbing it down too much for them. (P5)

HCP barriers reported within the literature related to poor communication skills where the patient ideas are undermined or dismissed,^18^ limiting or withholding information.^18^ or appearing condescending to highly literate patients when providing information.^10^, ^23^, ^24^ RT‐reported barriers also include time constraints for interactions.^2^, ^9^ Table 7 shows RT‐related barriers from this research and suggested options for solutions.^9^, ^19^ While many solutions might appear to be common sense, they may not be obvious to less experienced RT staff using HL strategies with patients. Knowledge of barriers and solutions may improve RT awareness when implementing HL strategies with patients.

**Table 7:** Tips for addressing radiation therapist related barriers when using HL strategies with patients.

Radiation therapist related barriers	
Barrier	Option for a solution
Timing and location of patient questions	On a case‐by‐case basis, the radiation therapist (RT) is in control, to determine where or when is the best time or place to answer questions. Encourage questions during informational sessions or at the beginning or end of a procedure. Questions allow people to learn new content and determine their level of understanding. Invite questions. ‘what are your questions?’ Or ‘what questions do you have?’ open way of asking. When addressing the questions, check back in with the patient to determine if you have answered it correctly. There are no stupid questions, offer reassurance.^18^
Inexperienced RT staff unable to manage patient HL needs	RT needs to complete communication and HL training to determine correct patient management.
Finding the right words when simplifying technical jargon	Connect new pieces of information to previous information.^18^ such as during a simulation session where analogies of a CT scan is described like slicing bread.^9^
RTs intimidated by educational or professional status of patients	RTs will be the expert, use universal precautions, adapt language and explanation by patients’ level of language and questions. Be ready to become more technical quickly when explaining concepts.
Using the teach‐back method is time intensive	To be completely patient‐focussed takes time; time to provide clear explanations, build trust and rapport and check for understanding. Ensure appropriate time is allocated. Improved patient understanding may reduce future treatment times and repeated imaging long term.

## Limitations

Several limitations exist to this study including social desirability response bias given the researcher’s professional relationship with the participants. Volunteer bias may have resulted in more positive reporting of experiences. Insider and outsider positions of the researcher may present bias; however, bracketing documents were completed to negate this. Time from workshops to interview was three years and this may have had an impact on participants’ recall of concepts.

## Conclusion

Opportunities to explore how RTs use HL strategies revealed adaptive practice relevant to specific patient needs and encountered barriers. Outcomes from this study have prompted an improvement in facilitation of future workshops; more time and options for phrasing to practice HL strategies and addressing known barriers during workshops for preparation of the clinical environment. Future research in this area is to promote HL across all levels of organisations. The workshop design could be transferrable to allied health staff working with RT patients, such as nursing, dieticians and psycho‐oncologists. Adapting workshops for other allied health staff caring for radiotherapy patients provides cohesion and consistent messaging to the patient. Furthermore, investigating the perceptions of patients of varying levels of health literacy as recipients of the HL strategies and their impressions would also be of benefit and may further strengthen HCP motivation to employ them.

## Conflict of Interest

The authors declares no conflict of interest.

## Supporting information

Researcher memo regarding interviews
