# Supplementary material for: Lived experiences of radiation therapists using health literacy strategies with patients—A qualitative review using interpretative phenomenological analysis
Source: J Med Radiat Sci. 2022 Mar 14;69(3):318–26. doi: 10.1002/jmrs.574 (PMC9442295; doi:10.1002/jmrs.574)
Supplement: Supplementary file 1 — Researcher memo regarding interviews [file JMRS-69-318-s001.docx]

**Supporting Information**

**Researcher Memo regarding interviews**

**Journal of Medical Radiation Sciences**

**Researcher Memo regarding interviews**

Before the interviews….

MARCH 21: So the invitations to participate have been sent out with some prompting at both sites.

APRIL 2021: With not much engagement or responses with staff after the invitations being sent out except for the first response, I might struggle to actually get enough people to talk to. Maybe 3 years was too long ago for people to remember the workshops we ran.

Maybe there’s a chance they will get my workshops confused with the emotional cues training Belinda did most recently. I am going to a send a reminder out.

Wow, looking at the list of who participated in the workshops, there are only about 5 people who still work there. Hoping I can get a couple at least. XXXX has offered to promote the research opportunity for me and also is keen to be interviewed. I have two more people keen to participate. That makes 6 people which was the higher end of the target.

I have a total of 6 acceptances for interview.

I am hoping these interviews provide the research outcomes I am looking for. After finalising the last qualitative paper and reviewing the outcomes, I am excited to move to this part of the research as I felt I had perhaps uncovered a phenomena related to some hesitancy when employing the teach back method around, that was not really reported within the literature. Particularly as the teach back method is reported to be used in a universal precaution approach. I had also crafted the interview questions not to put too much of an emphasis on this noted barrier, but to ask enough of people as to their attitudes towards it. Of course, it is not the only outcome I am focussed on exploring more. I am keen to hear how staff use the elements of plain language and tech back and if they actually find them beneficial in their day to day interactions with their patients when assessing their levels of understanding. Hoping people are honest and not going to tell me want they think I want to hear.

In preparation for the interviews, the text book suggests providing the questions to participants beforehand to allow them some preparation time to gather their thoughts. Given it has been 3 years since the workshops I am really am unsure how much they will recall, so I thought this was a good idea. I make sure I print out the questions without the prompts, as I want to ask the prompts if I need to during the interview.

MAY:

Participant 1

So my first interview is done. I gave the questions over during the morning before the interview. The interview was scheduled during the afternoon. I thought this approach worked well, so people could still complete their clinical work but have time to prepare notes etc if they wanted to. My aim is to have this approach for the remaining interviews.

I was actually pretty nervous, not because of who I was interviewing, but also needed to check the device was recording ok, but also because this was a really important part of my research and I wanted to do a good job!

He really spoke a lot and gave a lot of detail. I think I got a bit excited in the moment and asked a couple of leading questions, but tried to stop when I realised. I was surprised by the level of detail he gave and also the lengths he mentioned he goes to make sure the patient understands what is going on. I was also surprised at the level of care he takes and how emotionally aware he is with his patients and their background and the way he describes his interactions.

PARTICIPANT 2

This interview went for an hour or so, very unexpected. She also gave lots of examples of words she uses, phrases she uses and details of really positive experiences and those that were not great. She was really honest too. She drew me into the interview. She also appeared to be really genuinely concerned for the patients she described. To the point where she said, she didn’t care how she looked or how long it took, she was prepared to invest time into the patient. Quite eye opening, but I did consider the long transcript was followed the hour long interview.

PARTICIPANT 3

The third interview was scheduled shortly after the second, on the same day. The interviews are emotionally and mentally tiring!! Maybe it depends on the interviewee. I didn’t expect that.

The 3^rd^ participant was interesting. He did surprise me at times with some of his insightfulness on patients. Some of his examples he gave were interesting. Appeared to be a health literacy advocate which was encouraging.

PARTICIPANT 4

This participant had some time to prepare some notes which allowed her to answer using her notes which I was fine with.

As the interview went on, she mentioned the type of patient demographic and this actually had not occurred to me before. I guess when you have similar types of patients, you get used to communicating to them in a certain way and you don’t keep your skills sharp to interact with all types of patients. This interview raised some points that the other 3 so far did not. I felt encouraged by this and they were also experiences I have never had or had not considered either.

PARTICIPANT 5

I was glad this lady did want to participate, as she had a lot of experience. It turns out she did not have the time to review the questions but was happy to proceed anyway. She raised the same patient demographic as P4 and its impact on her interactions. There was certainly information that would be useful.

PARTICIPANT 6

I completed this interview via Skype. This actually worked well. I set up the voice recorder next to my speaker to capture the audio. This interview was actually really insightful and she spoke really well. I was pleased she agreed to be part of the research.
